# Concomitant Acquired Hemophilia A and Acquired Von Willebrand Syndrome from Distinctive Autoantibodies: Case Report

**DOI:** 10.3390/hematolrep17050052

**Published:** 2025-10-16

**Authors:** Richard Yu, Mackenzie Bowman, Arnaud Bonnefoy, Paula James, Chai W. Phua

**Affiliations:** 1 Division of Hematology, Department of Medicine, Western University, 800 Commissioners Road East, London, ON N6A 5W9, Canada; 2 Division of Hematology, Department of Medicine, Queens University, 94 Stuart Street, Kingston, ON K7L 3N6, Canada; 3 Department of Hematology, University of Montreal, Montreal, QC H2X 0A9, Canada

**Keywords:** acquired hemophilia A, acquired von Willebrand syndrome, autoantibody, case report

## Abstract

**Background and Clinical Significance:** Acquired hemophilia A (AHA) and acquired von Willebrand syndrome (AVWS) are rare bleeding disorders that do not often present concurrently. Here, we report a coexisting AHA and AVWS case due to underlying autoantibodies to factor VIII (FVIII) and von Willebrand factor (VWF). **Case Presentation:** A patient with gastrointestinal bleeding and prolonged aPTT was diagnosed with AHA and AVWS. The patient was started on immunosuppression with prednisone, cyclophosphamide, and intravenous immunoglobulin, alongside recombinant porcine FVIII replacement, susoctocog alfa. AVWS reduced the half-life of susoctocog alfa, requiring more frequent dosing and laboratory monitoring until AVWS resolved. The patient had two further relapses; the most recent was treated with Rituximab, following which remission has been maintained. **Conclusions:** Given the potential therapeutic implications, VWF testing should be considered as part of the diagnostic workup for AHA.

## 1. Introduction

Acquired hemophilia A (AHA) is a rare disorder with a reported incidence of 1.0–1.5 cases per million [1]. It is due to autoantibodies that target factor VIII (FVIII), causing FVIII deficiency and, consequently, bleeding. Etiologies of AHA are varied; fifty percent are idiopathic. Other associated conditions include monoclonal gammopathy, lymphoproliferative disorder, autoimmune conditions (e.g., systemic lupus), or medications [2].

Acquired von Willebrand syndrome (AVWS) is another rare bleeding disorder characterized by deficiency and/or dysfunction of von Willebrand factor (VWF). Etiologies include autoantibodies that directly inhibit the activity of VWF or increase clearance from circulation [3], shear-stress proteolysis (e.g., aortic stenosis, cardiac devices) [4], and adsorption of VWF onto cells that express the VWF receptor glycoprotein Ib (e.g., Wilm’s tumors) [5]. A single-center Mayo Clinic study estimated a prevalence of 0.04% (1 per 2500 patients) [6]. 

We present a case of concomitant AHA and AVWS, driven by the production of two autoantibodies with distinct epitope specificities and their significant clinical implications.

## 2. Detailed Case Description

A 73-year-old Caucasian female with a history of stroke, ST-elevation myocardial infarction (STEMI) on aspirin, and collagenous colitis presented to the hospital in 2023 after one week of melena stools with chest pain from demand ischemia. Her hemoglobin was low at 49 g/L, necessitating transfusions of 3 units of packed red blood cells (PRBCs). Aside from pallor, her physical exam was largely unremarkable, with no signs of gingival bleeding, petechiae, or hemarthrosis. Further bloodwork with reference ranges is summarized in Table 1. An urgent upper endoscopy revealed a small, non-bleeding gastric erosion, while colonoscopy identified numerous bleeding angioectasias that required argon plasma coagulation. 

Hematology was consulted for an isolated prolonged activated partial thromboplastin time (aPTT) of 64 s (reference: 20–29 s). Testing confirmed a diagnosis of AHA, with FVIII activity <0.01 IU/mL (reference: 0.50–2.00 IU/mL) and a FVIII inhibitor level of 28.8 BU (reference: <0.6 BU). Unexpectedly, both VWF antigen (VWF:Ag) and VWF activity (VWF:GPIbM) were found to be low at 0.12 IU/mL (reference: 0.50–2.00 IU/mL) and <0.15 IU/mL (reference: 0.48–1.73 IU/mL), respectively. The VWF:GPIbM/VWF:Ag ratio was not calculated due to the VWF activity being below the detection limit. VWF testing was pursued reflexively as per institutional protocol to further investigate prolonged aPTT and to elucidate the cause for low FVIII activity [7]. These results confirm the diagnosis of AHA along with von Willebrand Factor deficiency. With no personal or family history of a bleeding disorder, we considered the possibility of both AHA and AVWS.

To manage the active bleeding, IV recombinant porcine FVIII, susoctocog alfa, (Obizur^®^, Takeda) was administered. An initial dose of 100 U/kg IV resulted in an FVIII activity recovery to 0.84 IU/mL. However, due to the low levels of von Willebrand Factor, the estimated half-life of susoctocog alfa was short, measuring approximately two hours. Given the absence of clear causes for AVWS on physical exam and investigations, we suspected immune-mediated AVWS. Therefore, we administered intravenous immunoglobulins (IVIG) at a dose of 1 g/kg and initiated oral cyclophosphamide and prednisone, each at 1 mg/kg.

**Figure 1:**
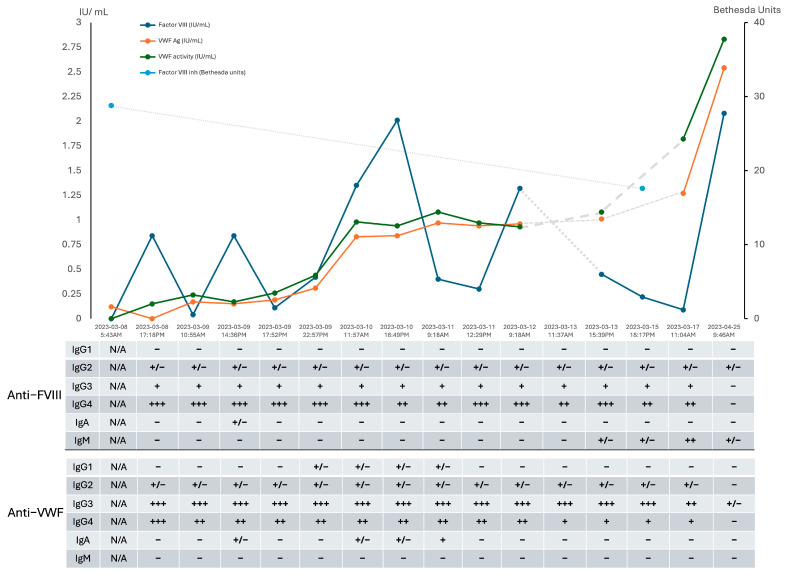
Timeline of the patient’s clinical course and associated diagnostic investigations shown are FVIII activity levels (blue), VWF Ag levels (orange), VWF activity (GPIbM assay) (green) and FVIII inhibitor level (teal) as a function of the patient’s clinical timeline. ELISA assessing for the presence of autoantibodies are correlated to the clinical dates. Dotted lines represent anticipated trends during periods without laboratory measurements. FVIII inhibitor testing was no longer indicated after 15 March 2023, as FVIII activity demonstrated a consistent and appropriate rise.

Approximately 24 h after IVIG administration, VWF testing showed rapid improvements in both VWF antigen and activity, suggesting a positive response to immunomodulation with IVIG (Figure 1). By 48 h after the initial IVIG dose, VWF levels had normalized, which led to an improvement in the half-life of susoctocog alfa to approximately 10 h in the presence of endogenous VWF, which protects FVIII from premature clearance.

Further workup with an echocardiogram showed aortic sclerosis without stenosis, with a negative bubble study. The CT body and PET-CT were not suggestive of malignancy. Extensive evaluation for potential underlying etiologies, including rheumatologic disorders (rheumatoid factor, ANCAs), occult malignancy (SPEP with immunofixation, CT/PET-CT imaging), valvular disease associated with aVWS (echocardiography), infectious triggers (HIV, hepatitis B, hepatitis C, COVID-19), and medication exposures, was negative. Given that approximately half of acquired hemophilia A cases are reported as idiopathic, the inhibitor development in this case is most likely idiopathic as well.

Additional laboratory testing was sent to a reference laboratory to review VWF Propeptide Antigen (VWFpp) and VWF Inhibitor Screen. These samples were drawn about 30 h after admission and post-IVIG dose with corresponding local laboratory testing showing VWF:Ag of 0.15 IU/mL and VWF:GPIbM of 0.17 IU/mL. The reference laboratory reported a VWF:GPIbM of 0.13 IU/mL and a VWFpp of 1.3 IU/mL. The ratio of VWFpp/VWF GP1bM activity was elevated at 10, suggesting increased clearance. The VWF inhibitor screen with the VWF GPIbM mixing study was negative. However, antibodies causing clearance without inhibition of function will not be detected by the assay.

The patient responded positively to treatment, with no further gastrointestinal bleeding, and was discharged after 12 days in the hospital. During her stay, she received transfusions of three units of packed red blood cells (pRBCs) and a total of 91,500 units of susoctocog alfa (approximately 817 U/kg) over ten days of exposure.

We retrospectively tested stored serial plasma samples using Enzyme-Linked Immunosorbent Assay (ELISA), which revealed the presence of polyclonal anti-FVIII antibodies (predominantly IgG-4 subtype) and anti-VWF antibodies (predominantly IgG-3 subtype) (Figure 1). Full-length recombinant factor VIII octocog alfa (Advate^®^, Takeda; final concentration: 4 IU/mL) and recombinant His-tagged von Willebrand factor (Creative BioMart, New York, USA; final concentration: 2 µg/mL) as target antigens were used. There was a clear inverse relationship between the optical density (OD) on ELISA and the improving VWF profile and FVIII activity, consistent with a response to therapy. This finding confirms the immune-mediated AVWS and AHA diagnoses. ELISA results were interpreted relative to the mean OD of negative controls plus three standard deviations. Samples below this threshold were considered negative (–); borderline (+/–) if above the threshold but <0.1; weakly positive (+) if ≥0.1 to <0.4; moderately positive (++) if ≥0.4 to <0.8; and strongly positive (+++) if ≥0.8. Further ELISA testing with different VWF concentrates antihemophilic factor/von Willebrand factor complex (human) [Humate-P^®^, CSL Behring], von Willebrand factor/factor VIII complex (human) [Wilate^®^, Octapharma], and recombinant von Willebrand factor (rVWF; Vonvendi^®^, Takeda) demonstrated binding of anti-VWF IgG antibodies to all products, with positivity defined as an optical density (OD) at 490 nm more than three standard deviations above the mean OD of six normal plasma samples. Notably, the recombinant VWF concentrate lacks FVIII, whereas the plasma derived VWF complexes contains both VWF and FVIII. Given the distinct polyclonal IgG patterns and the specificity of the IgG autoantibodies toward rVWF, we conclude that two distinct autoantibodies are responsible for the following: an anti-FVIII inhibitor consistent with AHA and an anti-VWF inhibitor consistent with immune-mediated AVWS.

At 4 months, the patient presented with gingival bleeding, requiring hospital admission. Further testing confirmed a relapse of AHA without AVWS relapse. FVIII activity was 0.08 IU/mL, with an FVIII inhibitor level of 8.8 BU, while the VWF profile remained within normal (VWF:Ag 1.21 IU/mL, VWF:GPIbM 1.12 IU/mL). Rechallenge with prednisone monotherapy led to a second complete response.

At 7 months, the patient experienced a partial relapse of AHA, discovered incidentally when she presented with Escherichia coli bacteremia and an isolated prolonged aPTT of 34 s. FVIII activity was mildly reduced at 0.37 IU/mL, a significant drop from 2.04 IU/mL measured three weeks earlier. The VWF profile was elevated (VWF:Ac 2.82 IU/mL, VWF:GPIbM 2.55 IU/mL), reflecting its role as an acute phase reactant. Immunosuppressive therapy with Rituximab (375 mg/m^2^ IV weekly for 4 weeks) and a short course of prednisone was administered. It is likely that early identification of the relapse prevented a full immune attack that could have further inhibited FVIII activity and led to bleeding.

At her last follow-up, 15 months after her initial diagnosis, the patient remained in remission, with normalization of both FVIII activity and her VWF profile. 

## 3. Discussion

Concomitant AHA and immune-mediated AVWS are exceedingly rare, with only one other case reported in the literature [7]. Extensive evaluation for potential underlying etiologies, including rheumatologic disorders (rheumatoid factor, ANCAs), occult malignancy (SPEP with immunofixation, CT/PET-CT imaging), valvular disease associated with aVWS (echocardiography), infectious triggers (HIV, hepatitis B, hepatitis C, COVID-19), and medication exposures, was negative. Given that approximately half of acquired hemophilia A cases are reported as idiopathic, the inhibitor development in this case is most likely idiopathic. The differential relapse patterns of AHA and AVWS further support the interpretation that these represent two distinct autoimmune processes rather than a single shared pathway. Although no underlying etiology was identified despite extensive workup, the autoantibodies were transient and reversed with treatment. This raises the hypothesis of a single, immune trigger, although the precise mechanism remains speculative.

VWF testing was pursued reflexively as per institutional protocol to investigate prolonged aPTT in a bleeding patient and to rule out VWD as a cause for low FVIII activity [8]. We recognize that this approach is not standard practice in most settings; however, given that FVIII pharmacokinetics depends on VWF, we recommend routine assessment of VWF when evaluating patients with isolated prolonged aPTT for suspected AHA, as this can have significant therapeutic implications. In this case, the low VWF levels led to a shortened half-life (~2 h) of susoctocog alfa, necessitating more frequent dosing initially. This improved to 10 h as VWF recovered, causing significant variations in FVIII activity peaks and troughs. Our hemostasis lab’s restricted hours contributed to delays in testing and treatment adjustments, highlighting the critical importance of adequate hemostatic laboratory support, especially during active bleeds to assist with susoctocog alfa dose adjustment; however, access to specialized hemostatic laboratory testing can be challenging [9].

Plasma-derived concentrates containing both FVIII and VWF were not attempted in this case due to the rapid resolution of the immune-mediated AVWS after IVIG. Whether the patient would have responded to VWF concentrate remains uncertain. Theoretically, the antibodies to VWF could lead to increased VWF clearance, and while the patient might initially respond to VWF concentrate, this response would likely be unsustainable if the antibody persists and continues to cause increased clearance. It is important to note that FVIII replacement therapy with plasma-derived FVIII has limited efficacy when the FVIII inhibitor titer exceeds 5 BU [10].

FVIII inhibitors were detected with the Nijmegen-Bethesda Assay [11], while a modified Bethesda method assessed VWF activity inhibition [12]. These tests lack sensitivity for detecting anti-VWF antibodies that target VWF clearance.

ELISA tests identified polyclonal IgG responsible for both AHA and immune-mediated AVWS, consistent with literature findings that show IgG4 is commonly involved in both disorders [13]. Figure 1 shows increased IgA and IgG1 antibodies, likely related to IVIG exposure. Pathogenic IgG2, IgG3, and IgG4 antibodies exhibited similar signal strength before and immediately after IVIG administration, then decreased over time due to immunosuppressive therapy.

IVIG is the recommended treatment for IgG monoclonal gammopathy associated with AVWS [5]. In this case, serum immunofixation did not demonstrate paraproteinemia. The therapeutic response observed in the immune-mediated AVWS appears to be temporally related to IVIG administration, though an early response to cyclophosphamide and prednisone cannot be excluded. Emicizumab, a humanized bispecific monoclonal antibody bridging activated FIX and FX (Hemlibra^®^, Genentech/Roche), may have a therapeutic role in future relapses, as it has demonstrated efficacy in treating AHA and as prophylaxis in severe type 3 VWD [14].

## 4. Conclusions

This is a rare case of concomitant AHA and immune-mediated AVWS, and to our knowledge, it is only the second such case reported in the literature [7]. The patient responded well to immunosuppressive therapy with cyclophosphamide and prednisone, with the immune-mediated AVWS showing a prompt response to IVIG. Recombinant porcine FVIII, susoctocog alfa, was effective for acute bleed management. AVWS led to a shortened half-life of susoctocog alfa, emphasizing the need to assess VWF levels and ensure frequent laboratory monitoring with specialized hemostasis support when diagnosing and managing AHA.

## Figures and Tables

**Table 1:** Summary of baseline and pertinent laboratory investigations.

Test	Value	Units	Reference Range
**Hematology**			
Hemoglobin	49	g/L	115–160
Platelets	263	×10^9^/L	150–400
Leukocytes	6.3	×10^9^/L	4–10
**Coagulation**			
INR	1.0	−	0.9–1.1
aPTT	64	sec	20–29
FVIII Activity	<0.01	IU/mL	0.5–1.5
FVIII Inhibitor	28.8	BU	<0.6
FIX Activity	1.57	IU/mL	0.5–2.0
FXI Activity	0.75	IU/mL	0.5–2.0
**VWF Studies**			0.5–2.0
VWF antigen (VWF Ag)	0.12	IU/mL	0.5–2.0
VWF Activity (VWF:GP1bM)	<0.15	IU/mL	0.48–1.73
**Reference Laboratory Testing**			
VWF Propeptide (VWFpp)	1.3	IU/mL	0.56–1.4
VWF Activity (VWF:GP1bM)	0.13	IU/mL	0.52–1.8
VWF GP1bM Mixing study	Negative	−	-
VWF Multimers	Faint bands, no preferential loss of HMW multimers	−	-

Other baseline investigations, including biochemical, hepatic, renal, viral, and autoimmune studies (serum electrolytes, renal and liver function tests, ferritin, haptoglobin, antinuclear antibodies, anti-neutrophil cytoplasmic antibodies, rheumatoid factor, serum protein electrophoresis with immunofixation, serum free light chains, and serological testing for hepatitis B, hepatitis C, COVID-19, and human immunodeficiency virus), were all within normal limits. Abbreviations: aPTT, activated partial thromboplastin time; BU, Bethesda units; HMW, high molecular weight; INR, international normalized ratio; VWF, von Willebrand factor; VWF:GPIbM, von Willebrand factor activity measured by glycoprotein Ib mutant assay.

## Data Availability

The original contributions presented in this study are included in the article. Further inquiries can be directed to the corresponding author.

## Abbreviations

The following abbreviations are used in this manuscript:

AHA	Acquired Hemophilia A
ANCA	Anti-Neutrophil Cytoplasmic Antibodies
aPTT	Activated Partial Thromboplastin Time
AVWS	Acquired von Willebrand Syndrome
BU	Bethesda Unit
COVID-19	Coronavirus Disease
CT	Computed Tomography
ELISA	Enzyme-Linked Immunosorbent Assay
FVIII	Factor VIII
FIX	Factor IX
FXI	Factor XI
GPIb	Glycoprotein Ib
HIV	Human Immunodeficiency Virus
HMW	High Molecular Weight
IVIG	Intravenous Immunoglobulins
PRBC	Packed Red Blood Cells
STEMI	ST-elevation Myocardial Infarction
VWFpp	VWF Propeptide Antigen
